# Methyl 2,2-bis­(2,4-dinitro­phen­yl)ethano­ate

**DOI:** 10.1107/S1600536811036440

**Published:** 2011-09-30

**Authors:** D. Kalaivani, R. Malarvizhi, M. Nethaji, S. Rajeswari

**Affiliations:** a PG and Research Department of Chemistry, Seethalakshmi Ramaswami College, Tiruchirappalli 620 002, Tamil Nadu, India; b Department of Inorganic & Physical Chemistry, Indian Institute of Science, Bangalore 560 012, India; c Department of Chemistry, Faculty of Engineering and Technology, SRM University, Kattankulathur 603 203, Tamil Nadu, India

## Abstract

In the title compound, C_15_H_10_N_4_O_10_, the dihedral angle between the aromatic rings is 89.05 (16)°. One O atom of one of the nitro groups is disordered over two sites in a 0.70:0.30 ratio. In the crystal, the mol­ecules are linked by weak C—H⋯O inter­actions.

## Related literature

For related structures, see: Chudek *et al.* (1989 ▶); Ertas *et al.* (1998 ▶). For background to the uses of eth­yl/methyl 2,2-bis­(2,4-dinitro­phen­yl)ethano­ates, see: Hu (2005 ▶); Kawai & Watanabe (2002 ▶); Liu *et al.* (2009 ▶). For further synthetic details, see: McIvor & Miller (1965 ▶).
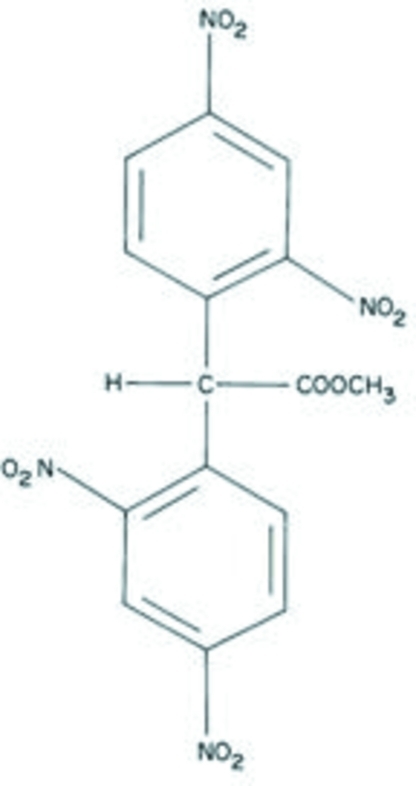


## Experimental

### 

#### Crystal data

C_15_H_10_N_4_O_10_
*M* *_r_* = 406.27
Monoclinic, 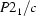

*a* = 9.812 (5) Å
*b* = 10.974 (6) Å
*c* = 16.025 (9) Å
β = 91.589 (10)°
*V* = 1724.8 (16) Å^3^
*Z* = 4
Mo *K*α radiation
μ = 0.14 mm^−1^
*T* = 293 K
0.34 × 0.29 × 0.27 mm

#### Data collection

Bruker SMART APEX CCD diffractometer
Absorption correction: multi-scan (*SADABS*; Bruker, 2006 ▶) *T*_min_ = 0.955, *T*_max_ = 0.965
13533 measured reflections
3525 independent reflections
1981 reflections with *I* > 2σ(*I*)
*R*_int_ = 0.046

#### Refinement

*R*[*F* ^2^ > 2σ(*F* ^2^)] = 0.077
*wR*(*F* ^2^) = 0.293
*S* = 0.98
3525 reflections
262 parameters
H-atom parameters constrained
Δρ_max_ = 0.86 e Å^−3^
Δρ_min_ = −0.52 e Å^−3^

### 

Data collection: *APEX2* (Bruker, 2006 ▶); cell refinement: *SAINT* (Bruker, 2006 ▶); data reduction: *SAINT*; program(s) used to solve structure: *SHELXS97* (Sheldrick, 2008 ▶); program(s) used to refine structure: *SHELXL97* (Sheldrick, 2008 ▶); molecular graphics: *ORTEP*-III (Burnett & Johnson, 1996 ▶); software used to prepare material for publication: *SHELXL97*.

## Supplementary Material

Crystal structure: contains datablock(s) global, I. DOI: 10.1107/S1600536811036440/hb6396sup1.cif

Structure factors: contains datablock(s) I. DOI: 10.1107/S1600536811036440/hb6396Isup2.hkl

Supplementary material file. DOI: 10.1107/S1600536811036440/hb6396Isup3.cml

Additional supplementary materials:  crystallographic information; 3D view; checkCIF report

## Figures and Tables

**Table 1:** Hydrogen-bond geometry (Å, °)

*D*—H⋯*A*	*D*—H	H⋯*A*	*D*⋯*A*	*D*—H⋯*A*
C2—H2⋯O5^i^	0.98	2.43	3.301 (5)	149
C7—H7⋯O10^ii^	0.93	2.38	3.273 (5)	160
C15—H15*A*⋯O8^iii^	0.96	2.59	3.255 (5)	127
C15—H15*B*⋯O3^i^	0.96	2.41	3.216 (9)	141

Symmetry codes: (i) 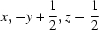; (ii) 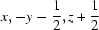; (iii) 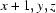.

**Fig. 1.**
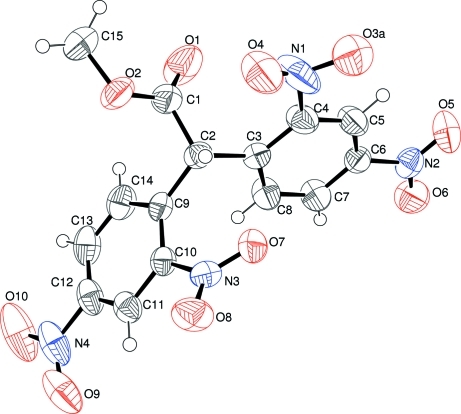
The molecular structure of (I) showing 50% displacement ellipsoids.

## supplementary crystallographic information

### Comment

Ethyl / methyl 2,2-bis(2,4-dinitrophenyl)ethanoates have been used in many analytical detections (Kawai & Watanabe, 2002) and for the preparation of photo degradable aqueous inks (Hu, 2005) and tailor's chalk (Liu *et al.*, 2009). Articles have also appeared on the structure of ethyl 2,2-bis(2,4-dinitrophenyl) ethanoate (Chudek *et al.*, 1989, Ertas *et al.*, 1998).

Ethyl 2,2-bis(2,4-dinitrophenyl)ethanoate has been synthesized (yield 32–71%) by mixing equimolar amounts of 1-chloro-2,4-dinitrobenzene and ethyl 2,4-dinitrophenylacetate in the presence of tripropylamine in dimethylformamide medium (McIvor and Miller, 1965). The same authors have also prepared methyl 2,2-bis(2,4-dinitrophenyl)ethanoate (title molecule) only in 32% yield by adopting the same procedure. In this article we report an efficient new one pot synthesis to prepare title molecule (scheme) in good yield (65–70%) with high purity. The acetyl group of methyl 3-oxobutanoate has cleaved off during the formation of the title molecule.

The *ORTEP* diagram of title molecule is presented in Figure 1. The packing of the molecules features a number of C—H···O hydrogen bonds (Table 1).

### Experimental

1-Chloro-2,4-dinitrobenzene (2 g, 0.01 mol) in absolute ethanol was mixed with methyl 3-oxobutanoate (1.2 g, 0.01 mol) in absolute ethanol. Triethylamine (5 g, 0.05 mol) was then added and the mixture was shaken well for 5 to 6 h. On standing pale yellow crystals come out from the solution after 15 days. The crystals were filtered and washed well with distilled water and dried. The dried crystals were powdered and washed with copious amount of ether to remove the unreacted reactants and then with little absolute alcohol. The crystals obtained after washing were recrystallized either from ethylacetate or chloroform to yield colourless blocks of the title compound (yield 65–70%: m.pt. 428 K).

### Refinement

Refinement of F^2^ against ALL reflections. The weighted *R*-factor *wR* and

goodness of fit S are based on F^2^, conventional *R*-factors *R* are based

on F, with F set to zero for negative F^2^. The threshold expression of

F^2^ > 2σ(F^2^) is used only for calculating *R*-factors(gt) *etc*. and is

not relevant to the choice of reflections for refinement. *R*-factors based

on F^2^ are statistically about twice as large as those based on F, and *R*-

factors based on ALL data will be even larger.

### Crystal data

**Table:**

C_15_H_10_N_4_O_10_	*F*(000) = 832
*M**_r_* = 406.27	*D*_x_ = 1.565 Mg m^−^^3^
Monoclinic, *P*2_1_/*c*	Mo *K*α radiation, λ = 0.71073 Å
*a* = 9.812 (5) Å	Cell parameters from 567 reflections
*b* = 10.974 (6) Å	θ = 2.5–26.0°
*c* = 16.025 (9) Å	µ = 0.14 mm^−^^1^
β = 91.589 (10)°	*T* = 293 K
*V* = 1724.8 (16) Å^3^	Block, colorless
*Z* = 4	0.34 × 0.29 × 0.27 mm

### Data collection

**Table:**

Bruker SMART APEX CCD diffractometer	3525 independent reflections
Radiation source: fine-focus sealed tube	1981 reflections with *I* > 2σ(*I*)
graphite	*R*_int_ = 0.046
Detector resolution: 0.3 pixels mm^-1^	θ_max_ = 26.4°, θ_min_ = 2.1°
ω scans	*h* = −12→12
Absorption correction: multi-scan (*SADABS*; Bruker, 2006)	*k* = −13→13
*T*_min_ = 0.955, *T*_max_ = 0.965	*l* = −20→20
13533 measured reflections	

### Refinement

**Table:**

Refinement on *F*^2^	Primary atom site location: structure-invariant direct methods
Least-squares matrix: full with fixed elements per cycle	Secondary atom site location: difference Fourier map
*R*[*F*^2^ > 2σ(*F*^2^)] = 0.077	Hydrogen site location: inferred from neighbouring sites
*wR*(*F*^2^) = 0.293	H-atom parameters constrained
*S* = 0.98	*w* = 1/[σ^2^(*F*_o_^2^) + (0.2*P*)^2^] where *P* = (*F*_o_^2^ + 2*F*_c_^2^)/3
3525 reflections	(Δ/σ)_max_ = 0.012
262 parameters	Δρ_max_ = 0.86 e Å^−^^3^
0 restraints	Δρ_min_ = −0.52 e Å^−^^3^

### Special details

**Table:**

Geometry. All s.u.'s (except the s.u. in the dihedral angle between two l.s. planes)are estimated using the full covariance matrix. The cell s.u.'s are takeninto account individually in the estimation of s.u.'s in distances, anglesand torsion angles; correlations between s.u.'s in cell parameters are onlyused when they are defined by crystal symmetry. An approximate (isotropic)treatment of cell s.u.'s is used for estimating s.u.'s involving l.s. planes.
Refinement. Refinement of *F*^2^ against ALL reflections. The weighted *R*-factor *wR* andgoodness of fit *S* are based on *F*^2^, conventional *R*-factors *R* are basedon *F*, with *F* set to zero for negative *F*^2^. The threshold expression of*F*^2^ > 2σ(*F*^2^) is used only for calculating *R*-factors(gt) *etc*. and isnot relevant to the choice of reflections for refinement. *R*-factors basedon *F*^2^ are statistically about twice as large as those based on *F*, and *R*-factors based on ALL data will be even larger.

### Fractional atomic coordinates and isotropic or equivalent isotropic displacement parameters (Å^2^)

**Table:**

	*x*	*y*	*z*	*U*_iso_*/*U*_eq_	Occ. (<1)
O1	0.5549 (3)	0.1001 (4)	0.33068 (19)	0.1017 (12)	
O2	0.5029 (2)	0.1801 (3)	0.20813 (16)	0.0676 (8)	
O3	0.5184 (7)	0.3122 (6)	0.4951 (5)	0.136 (2)*	0.7
O3A	0.4078 (11)	0.3806 (10)	0.5064 (7)	0.086 (3)*	0.3
O4	0.4263 (4)	0.3497 (3)	0.38083 (18)	0.0951 (10)	
O5	0.2382 (4)	0.1011 (2)	0.69206 (16)	0.0784 (9)	
O6	0.1291 (3)	−0.0550 (3)	0.64934 (17)	0.0822 (9)	
O7	0.0686 (2)	0.2483 (2)	0.30932 (14)	0.0568 (6)	
O8	−0.0449 (3)	0.2648 (3)	0.19403 (17)	0.0695 (8)	
O9	−0.0984 (4)	−0.1013 (3)	0.03896 (17)	0.0908 (10)	
O10	0.0800 (5)	−0.2084 (3)	0.0167 (2)	0.1186 (14)	
N1	0.4152 (5)	0.3015 (4)	0.4429 (2)	0.1069 (15)	
N2	0.1982 (3)	0.0338 (3)	0.63757 (17)	0.0529 (7)	
N3	0.0397 (3)	0.2146 (2)	0.23940 (17)	0.0455 (7)	
N4	0.0206 (4)	−0.1288 (3)	0.05241 (18)	0.0742 (11)	
C1	0.4734 (4)	0.1400 (4)	0.2825 (2)	0.0544 (9)	
C2	0.3221 (3)	0.1506 (3)	0.29780 (19)	0.0424 (8)	
H2	0.2969	0.2361	0.2889	0.051*	
C3	0.2927 (3)	0.1203 (3)	0.38819 (19)	0.0420 (8)	
C4	0.3366 (4)	0.1903 (3)	0.4556 (2)	0.0557 (9)	
C5	0.3089 (4)	0.1621 (3)	0.5366 (2)	0.0565 (10)	
H5	0.3412	0.2108	0.5804	0.068*	
C6	0.2338 (3)	0.0622 (3)	0.55141 (19)	0.0444 (8)	
C7	0.1878 (4)	−0.0127 (3)	0.4884 (2)	0.0555 (9)	
H7	0.1371	−0.0819	0.4999	0.067*	
C8	0.2183 (4)	0.0169 (3)	0.4073 (2)	0.0548 (9)	
H8	0.1881	−0.0339	0.3642	0.066*	
C9	0.2407 (3)	0.0761 (3)	0.23477 (18)	0.0420 (7)	
C10	0.1093 (3)	0.1079 (3)	0.20720 (18)	0.0399 (7)	
C11	0.0366 (4)	0.0419 (3)	0.14770 (19)	0.0485 (8)	
H11	−0.0506	0.0654	0.1302	0.058*	
C12	0.0968 (4)	−0.0586 (3)	0.1154 (2)	0.0547 (10)	
C13	0.2250 (5)	−0.0943 (3)	0.1401 (2)	0.0610 (11)	
H13	0.2645	−0.1632	0.1171	0.073*	
C14	0.2945 (4)	−0.0269 (3)	0.1994 (2)	0.0586 (10)	
H14	0.3815	−0.0517	0.2164	0.07*	
C15	0.6420 (4)	0.1669 (5)	0.1825 (3)	0.0790 (13)	
H15A	0.7002	0.2184	0.2163	0.119*	
H15B	0.6483	0.19	0.125	0.119*	
H15C	0.67	0.0836	0.1892	0.119*	

### Atomic displacement parameters (Å^2^)

**Table:**

	*U*^11^	*U*^22^	*U*^33^	*U*^12^	*U*^13^	*U*^23^
O1	0.0536 (16)	0.175 (3)	0.0770 (19)	0.039 (2)	0.0044 (14)	0.038 (2)
O2	0.0451 (13)	0.099 (2)	0.0595 (15)	−0.0030 (14)	0.0116 (11)	0.0197 (14)
O4	0.130 (3)	0.091 (2)	0.0644 (17)	−0.057 (2)	0.0008 (17)	0.0107 (15)
O5	0.114 (2)	0.0730 (19)	0.0486 (14)	0.0012 (17)	0.0103 (14)	−0.0108 (13)
O6	0.0809 (19)	0.104 (2)	0.0625 (16)	−0.0308 (17)	0.0112 (14)	0.0161 (15)
O7	0.0572 (14)	0.0635 (15)	0.0499 (12)	0.0047 (12)	0.0041 (11)	−0.0124 (11)
O8	0.0556 (15)	0.0680 (17)	0.0838 (17)	0.0205 (13)	−0.0170 (13)	−0.0031 (14)
O9	0.142 (3)	0.0722 (19)	0.0568 (15)	−0.0440 (19)	−0.0298 (16)	0.0104 (13)
O10	0.233 (4)	0.0577 (18)	0.0638 (18)	0.013 (2)	−0.018 (2)	−0.0165 (15)
N1	0.144 (3)	0.124 (3)	0.0516 (19)	−0.089 (3)	−0.020 (2)	0.010 (2)
N2	0.0522 (17)	0.0567 (18)	0.0501 (15)	0.0065 (14)	0.0086 (13)	0.0082 (13)
N3	0.0356 (13)	0.0472 (16)	0.0535 (15)	−0.0013 (12)	0.0002 (11)	−0.0005 (12)
N4	0.136 (3)	0.0434 (17)	0.0423 (16)	−0.0151 (19)	−0.0114 (17)	0.0084 (13)
C1	0.0470 (19)	0.065 (2)	0.0512 (19)	0.0017 (18)	−0.0009 (16)	0.0027 (17)
C2	0.0379 (16)	0.0461 (18)	0.0433 (16)	−0.0005 (14)	0.0026 (13)	0.0064 (14)
C3	0.0373 (16)	0.0432 (18)	0.0455 (16)	0.0003 (14)	0.0018 (13)	0.0016 (14)
C4	0.060 (2)	0.058 (2)	0.0494 (18)	−0.0170 (18)	−0.0038 (16)	0.0050 (16)
C5	0.064 (2)	0.062 (2)	0.0436 (18)	−0.0139 (19)	−0.0057 (16)	−0.0023 (16)
C6	0.0417 (17)	0.0492 (19)	0.0425 (16)	0.0015 (15)	0.0021 (13)	0.0025 (14)
C7	0.070 (2)	0.0450 (19)	0.0515 (19)	−0.0121 (18)	0.0027 (17)	0.0065 (16)
C8	0.072 (2)	0.046 (2)	0.0463 (18)	−0.0109 (18)	0.0038 (16)	0.0014 (15)
C9	0.0449 (17)	0.0445 (18)	0.0370 (15)	−0.0010 (15)	0.0097 (13)	0.0024 (13)
C10	0.0456 (17)	0.0385 (17)	0.0360 (15)	−0.0007 (14)	0.0049 (13)	0.0029 (12)
C11	0.059 (2)	0.0480 (19)	0.0385 (16)	−0.0071 (17)	−0.0011 (14)	0.0073 (14)
C12	0.084 (3)	0.0434 (19)	0.0364 (16)	−0.0099 (19)	0.0033 (16)	0.0040 (14)
C13	0.090 (3)	0.042 (2)	0.052 (2)	0.008 (2)	0.018 (2)	−0.0016 (16)
C14	0.063 (2)	0.053 (2)	0.060 (2)	0.0132 (19)	0.0123 (18)	−0.0016 (18)
C15	0.052 (2)	0.111 (4)	0.075 (3)	−0.007 (2)	0.016 (2)	−0.001 (3)

### Geometric parameters (Å, °)

**Table:**

O1—C1	1.180 (4)	C3—C4	1.385 (5)
O2—C1	1.311 (4)	C3—C8	1.387 (5)
O2—C15	1.443 (5)	C4—C5	1.369 (5)
O3—N1	1.302 (7)	C5—C6	1.346 (5)
O3—O3A	1.336 (12)	C5—H5	0.9300
O3A—N1	1.341 (11)	C6—C7	1.369 (5)
O4—N1	1.134 (4)	C7—C8	1.380 (5)
O5—N2	1.201 (3)	C7—H7	0.9300
O6—N2	1.206 (3)	C8—H8	0.9300
O7—N3	1.206 (3)	C9—C14	1.376 (5)
O8—N3	1.220 (3)	C9—C10	1.396 (5)
O9—N4	1.219 (4)	C10—C11	1.380 (4)
O10—N4	1.204 (4)	C11—C12	1.360 (5)
N1—C4	1.460 (5)	C11—H11	0.9300
N2—C6	1.467 (4)	C12—C13	1.365 (6)
N3—C10	1.457 (4)	C13—C14	1.371 (5)
N4—C12	1.459 (5)	C13—H13	0.9300
C1—C2	1.516 (5)	C14—H14	0.9300
C2—C9	1.510 (4)	C15—H15A	0.9600
C2—C3	1.522 (4)	C15—H15B	0.9600
C2—H2	0.9800	C15—H15C	0.9600
			
C1—O2—C15	117.4 (3)	C4—C5—H5	120.8
N1—O3—O3A	61.1 (6)	C5—C6—C7	122.0 (3)
O3—O3A—N1	58.2 (6)	C5—C6—N2	119.0 (3)
O4—N1—O3	115.4 (5)	C7—C6—N2	119.0 (3)
O4—N1—O3A	111.8 (6)	C6—C7—C8	118.5 (3)
O3—N1—O3A	60.7 (6)	C6—C7—H7	120.7
O4—N1—C4	125.2 (3)	C8—C7—H7	120.7
O3—N1—C4	113.0 (5)	C7—C8—C3	121.9 (3)
O3A—N1—C4	113.3 (6)	C7—C8—H8	119.0
O5—N2—O6	123.9 (2)	C3—C8—H8	119.0
O5—N2—C6	118.2 (3)	C14—C9—C10	115.9 (3)
O6—N2—C6	117.9 (3)	C14—C9—C2	121.2 (3)
O7—N3—O8	123.7 (2)	C10—C9—C2	122.9 (3)
O7—N3—C10	118.4 (2)	C11—C10—C9	122.9 (3)
O8—N3—C10	118.0 (2)	C11—C10—N3	115.4 (3)
O10—N4—O9	124.7 (3)	C9—C10—N3	121.8 (3)
O10—N4—C12	117.8 (3)	C12—C11—C10	117.8 (3)
O9—N4—C12	117.5 (3)	C12—C11—H11	121.1
O1—C1—O2	123.8 (3)	C10—C11—H11	121.1
O1—C1—C2	124.9 (3)	C11—C12—C13	121.9 (3)
O2—C1—C2	111.3 (3)	C11—C12—N4	118.1 (4)
C9—C2—C1	110.6 (3)	C13—C12—N4	120.0 (3)
C9—C2—C3	114.1 (3)	C12—C13—C14	118.8 (3)
C1—C2—C3	110.5 (3)	C12—C13—H13	120.6
C9—C2—H2	107.1	C14—C13—H13	120.6
C1—C2—H2	107.1	C13—C14—C9	122.7 (4)
C3—C2—H2	107.1	C13—C14—H14	118.7
C4—C3—C8	115.8 (3)	C9—C14—H14	118.7
C4—C3—C2	124.0 (3)	O2—C15—H15A	109.5
C8—C3—C2	120.2 (3)	O2—C15—H15B	109.5
C5—C4—C3	123.3 (3)	H15A—C15—H15B	109.5
C5—C4—N1	116.2 (3)	O2—C15—H15C	109.5
C3—C4—N1	120.5 (3)	H15A—C15—H15C	109.5
C6—C5—C4	118.4 (3)	H15B—C15—H15C	109.5
C6—C5—H5	120.8		
			
O3A—O3—N1—O4	101.8 (7)	O6—N2—C6—C7	−0.1 (4)
O3A—O3—N1—C4	−104.7 (7)	C5—C6—C7—C8	−0.9 (6)
O3—O3A—N1—O4	−107.8 (6)	N2—C6—C7—C8	178.0 (3)
O3—O3A—N1—C4	104.2 (6)	C6—C7—C8—C3	−0.6 (6)
C15—O2—C1—O1	4.1 (6)	C4—C3—C8—C7	1.3 (5)
C15—O2—C1—C2	−175.4 (3)	C2—C3—C8—C7	−179.0 (3)
O1—C1—C2—C9	−118.9 (4)	C1—C2—C9—C14	28.9 (4)
O2—C1—C2—C9	60.5 (4)	C3—C2—C9—C14	−96.4 (4)
O1—C1—C2—C3	8.4 (6)	C1—C2—C9—C10	−149.5 (3)
O2—C1—C2—C3	−172.2 (3)	C3—C2—C9—C10	85.2 (4)
C9—C2—C3—C4	−168.3 (3)	C14—C9—C10—C11	−0.5 (5)
C1—C2—C3—C4	66.4 (4)	C2—C9—C10—C11	177.9 (3)
C9—C2—C3—C8	12.1 (4)	C14—C9—C10—N3	179.9 (3)
C1—C2—C3—C8	−113.3 (4)	C2—C9—C10—N3	−1.6 (5)
C8—C3—C4—C5	−0.5 (6)	O7—N3—C10—C11	152.1 (3)
C2—C3—C4—C5	179.8 (3)	O8—N3—C10—C11	−27.7 (4)
C8—C3—C4—N1	−179.7 (4)	O7—N3—C10—C9	−28.3 (4)
C2—C3—C4—N1	0.6 (6)	O8—N3—C10—C9	151.9 (3)
O4—N1—C4—C5	−165.2 (5)	C9—C10—C11—C12	0.4 (5)
O3—N1—C4—C5	44.4 (6)	N3—C10—C11—C12	179.9 (3)
O3A—N1—C4—C5	−22.2 (7)	C10—C11—C12—C13	−0.2 (5)
O4—N1—C4—C3	14.1 (7)	C10—C11—C12—N4	−179.9 (3)
O3—N1—C4—C3	−136.3 (5)	O10—N4—C12—C11	171.1 (3)
O3A—N1—C4—C3	157.0 (6)	O9—N4—C12—C11	−8.0 (4)
C3—C4—C5—C6	−0.9 (6)	O10—N4—C12—C13	−8.7 (5)
N1—C4—C5—C6	178.4 (4)	O9—N4—C12—C13	172.2 (3)
C4—C5—C6—C7	1.6 (6)	C11—C12—C13—C14	0.2 (6)
C4—C5—C6—N2	−177.3 (3)	N4—C12—C13—C14	180.0 (3)
O5—N2—C6—C5	−0.3 (4)	C12—C13—C14—C9	−0.5 (6)
O6—N2—C6—C5	178.8 (3)	C10—C9—C14—C13	0.6 (5)
O5—N2—C6—C7	−179.2 (3)	C2—C9—C14—C13	−177.9 (3)

### Hydrogen-bond geometry (Å, °)

**Table:**

*D*—H···*A*	*D*—H	H···*A*	*D*···*A*	*D*—H···*A*
C2—H2···O5^i^	0.98	2.43	3.301 (5)	149
C7—H7···O10^ii^	0.93	2.38	3.273 (5)	160
C15—H15A···O8^iii^	0.96	2.59	3.255 (5)	127
C15—H15B···O3^i^	0.96	2.41	3.216 (9)	141

Symmetry codes: (i) *x*, −*y*+1/2, *z*−1/2; (ii) *x*, −*y*−1/2, *z*+1/2; (iii) *x*+1, *y*, *z*.
